# Antibacterial Activity of *Rosmarinus officinalis* against Multidrug-Resistant Clinical Isolates and Meat-Borne Pathogens

**DOI:** 10.1155/2021/6677420

**Published:** 2021-04-29

**Authors:** Aseer Manilal, Kuzhunellil Raghavanpillai Sabu, Melat Woldemariam, Addis Aklilu, Gelila Biresaw, Tsegaye Yohanes, Mohammed Seid, Behailu Merdekios

**Affiliations:** ^1^Department of Medical Laboratory Sciences, College of Medicine and Health Sciences, Arba Minch University, Arba Minch, Ethiopia; ^2^Department of Chemistry, College of Life Sciences, Arba Minch University, Arba Minch, Ethiopia; ^3^Department of Public Health, College of Medicine and Health Sciences, Arba Minch University, Arba Minch, Ethiopia

## Abstract

**Background:**

In developing countries, the prevalence of bacterial infections is quite rampant due to several factors such as the HIV/AIDS pandemic, lack of hygiene, overcrowding, and resistance to conventional antimicrobials. Hence the use of plant-based antimicrobial agents could provide a low-cost alternative therapy. *Rosmarinus officinalis* is reputed as a medicinal plant in Ethiopia; however, its antibacterial activity against many of the clinical isolates remains overlooked.

**Methods:**

Tender foliage of *R. officinalis* was collected and extracted in ethanol (EtOH) and evaluated for their antimicrobial activity against ten multidrug-resistant (MDR) clinical isolates, human type culture pathogens, and meat-borne bacterial isolates by employing agar well diffusion assay.

**Results:**

EtOH extract of *R. officinalis* efficiently subdued the growth of all tested MDR clinical isolates in varying degrees. *Salmonella* sp. and *Staphylococcus aureus* were found to be the most sensitive clinical isolates. Likewise, it efficiently repressed the growth of meat-borne pathogens, particularly, *S. aureus* and *Salmonella* sp. showing its potentiality to be used as a natural antibacterial agent in the meat processing industry. The mechanism of antibiosis of plant extract against meat-borne pathogens is inferred to be bactericidal. Chemical constituents of the crude plant extract were analysed by Gas Chromatography-Mass Spectroscopy (GC-MS), Fourier Transform Infrared (FT-IR), and UV-visible spectroscopy showing genkwanin (26%), camphor (13%), endo-borneol (13%), alpha-terpineol (12%), and hydroxyhydrocaffeic acid (13%) as the major compounds.

**Conclusion:**

Overall results of the present study conclude that *R. officinalis* could be an excellent source of antimicrobial agents for the management of drug-resistant bacteria as well as meat-borne pathogens.

## 1. Introduction

Nowadays, MDR pathogens are becoming extremely common and inflict life-threatening infections in hospitals (nosocomial) and also at the community level. Bacteria possess a broad spectrum of genetic potency to transmit and acquire resistance to diverse types of drugs that are currently in use and any new synthetic antimicrobials introduced will become sooner or later ineffective [1]. Recently, WHO reviewed a list of priority bacterial pathogens belonging to 12 families that urgently need novel and effective antibiotics to deal with [2]. Above all, the medical fraternity is now warning that there will be a return to the preantibiotic era. Infections caused by MDR pathogens increase healthcare costs, length of stay in hospitals, morbidity, and mortality in both developed and developing countries [3]. Currently, at least 0.7 million die each year due to diseases caused by drug-resistant pathogens [4]. Treatment of infections caused by them has become extremely challenging for the physicians, particularly in African countries where a definitive therapy is only occasional [5].

The increasing prevalence of MDR organisms warrants an urgent search for novel antimicrobial agents and their development. Flora represents a gold mine of diverse varieties of bioactive secondary metabolites (including antimicrobials) and is in extensive use in traditional medicines worldwide. Some of these secondary metabolites offer avenues for developing inexpensive, safe, and effective antibiotics. It is important to note that bacteria are inept to easily develop resistance to the multiple and/or chemically complex phytochemicals present in plant extracts [6]. Therefore, studies related to the identification and utilization of safe and potent antimicrobials from plants have been supported by the WHO and they constitute the research priority of numerous groups, including ours. In Ethiopia, rural populations heavily rely on traditional medicines for the treatment of many infectious diseases. An estimate of the WHO depictured that 90% of Ethiopians are using traditional medicines for primary healthcare [7]. Many of these plants used in traditional medicines are without any scientific evidence of efficacy. Antimicrobial screening of plants is a stepping stone in the discovery of novel antimicrobial drugs. Therefore, it is quite relevant to identify and evaluate plants with antimicrobial potency.

*Rosmarinus officinalis* belongs to the family Lamiaceae, which is an aromatic evergreen shrub distributed throughout the world and extensive research work is being carried out on its phytochemical constituents [8, 9]. It is traditionally used in medicinal formulations, and also as an important spice, flavouring agent, and food preservative [10–12]. This plant is known to exhibit antitumor, antiviral, antibacterial, anti-inflammatory, antithrombotic, diuretic, antidiabetic, and antioxidant activities [12, 13]. *Rosmarinus officinalis* is one of the prioritized medicinal crops in Ethiopia and according to an ethnobotanical survey it is medicinally used all over the country [14]. In addition, the leaves of this plant find frequent application as a flavouring agent in local traditional meat dishes both cooked and raw. Results obtained from our previous study showed that the EtOH extract of *R. officinalis* has anti-methicillin resistant *S. auerus* activity [15]. To acquire more relevant data related to its antimicrobial activity, further comprehensive evaluations are imperative. A perusal of the literature showed that antimicrobial activities of *R. officinalis* against MDR clinical and food-borne isolates are scanty in an Ethiopian context. In this respect, the present study has been designed to evaluate the antimicrobial activities of *R. officinalis* against MDR clinical isolates associated with enteric, urinary tract, and wound infections. Besides, its activity was also inspected against a panel of meat-borne pathogens, because in Ethiopia, this plant finds widespread utility in meat dishes. Major phytoconstituents in the plant were analysed by GC-MS, FT-IR, and UV-visible spectroscopy.

## 2. Materials and Methods

### 2.1. Collection of *Rosmarinus officinalis* and Its Extraction

Field collection of fresh tender foliage of *Rosmarinus officinalis* (Voucher Specimen No. AMP11) was done from the local markets of Arba Minch town, Gamo Gofa zone of Ethiopia. The plant specimen was botanically authenticated as previously indicated [15]. It was identified to the genus and species level with the aid of an eminent plant taxonomist (Dr. Remesh Moochikkal, Jazan University, Kingdom of Saudi Arabia). Prior to extraction, the leaves were cleaned to remove dirt and associated debris. The cleaned samples were chopped into small pieces and stored at 2^o^C. The extraction procedure optimized in our previous study was employed, which found that EtOH is a solvent of suitable polarity [15]. Five *g* of leaves were threshed in a mortar by a pestle using the solvent at room temperature. The resultant extract was then ﬁltered through a Whatman No.1 filter paper and the residue was reextracted twice by using the same solvent. The total extract (final) was obtained by merging the ﬁltrates from all the three steps. It was then evaporated to dryness in a water bath at 50^o^C. The concentrated extract (about 5 mL) was collected in an airtight plastic vial and stored in the refrigerator until assay. The study protocol was approved by the Institutional Review Board of College of Medicine and Health Sciences, Arba Minch University.

### 2.2. Test Microorganisms

EtOH extract was tested against a panel of three type culture bacterial isolates, three meat-borne bacteria, and ten clinical isolates (Table 1). The pathogens with ATCC numbers *S. aureus* ATCC 25923, *Escherichia coli* ATCC 25922, and *Pseudomonas aeruginosa* ATCC 27853 were obtained from Ethiopian Public Health Institute (Table 1).

#### 2.2.1. Isolation and Identification of Meat-Borne Bacterial Isolates

Meat-borne bacterial pathogens were isolated from ready-to-eat meat purchased from local steak house restaurants and identified using the standard microbiological procedures [16–18]. Briefly mentioning, twenty-five grams of beef sample in 225 ml of sterile water was homogenized using a laboratory blender for 2 minutes and serially diluted (up to 10^−6^). To isolate *E. coli* and *S. aureus,* a loopful of inoculum from the one in 6 dilution of every sample was inoculated onto MacConkey agar and mannitol salt agar and incubated at 37°C for 24 hrs. In the case of *Salmonella* sp., after enrichment of different dilutions in selenite F broth, 0.1 ml aliquot was inoculated on xylose lysine deoxycholate agar and incubated at 37°C for 24 hrs. Pure colonies of respective bacterial isolates were identified based on colony morphology, Gram staining, and biochemical characteristics stated elsewhere [19].

#### 2.2.2. Isolation, Identification, and Antimicrobial Resistance Patterns of Clinical Isolates

Pathogenic bacteria were isolated from different clinical samples such as wound and throat swabs, urine, and stool collected from patients with complaints of wound and/or throat infections, possible urinary tract infection, and diarrhoea attending various departments of Arba Minch General Hospital which were received further by the Medical Microbiology and Parasitology Laboratory, College of Medicine and Health Sciences, Arba Minch University, Arba Minch. All the clinical samples were processed according to standard bacteriological methods and identified by conventional techniques [19]. Samples were plated out on a set of respective isolation media including MacConkey agar, sheep blood agar, and xylose lysine deoxycholate agar, cysteine lactose electrolyte deficient agar, and mannitol salt agar. The inoculated plates were incubated for 24 hrs at 37°C. For the isolation of fastidious bacteria, *Streptococcus pyogenes*, specimen are inoculated onto 5% sheep blood agar and incubated aerobically in the presence of 5–10%, v/v CO_2_. Likewise for *Campylobacter* sp., Campylobacter agar base with 10% sterile defibrinated sheep blood and rehydrated contents of Campylobacter Supplement-I (Blaser-Wang) (FD006) was used. Agar plates were incubated under microaerophilic conditions (5–10% O_2_ and 10% CO_2_ v/v concentration) at 42°C for 24–48 hrs. Following incubation, plates were inspected for bacterial growth. Pure cultures of respective bacterial isolates were subsequently subjected to species identification and confirmation. Macroscopic and microscopic analyses and biochemical and physiological characteristics of isolated bacteria were evaluated via standard laboratory methods as stated elsewhere [19]. Gram-positive isolates were identified using catalase and coagulase tests. Isolates of members of Enterobacteriaceae family were identified biochemically by means of a series of tests such as catalase, indole, citrate, urease, H_2_S production, methyl red, Voges-Proskauer, and triple sugar iron. Non-lactose fermenting Gram-negative bacteria were identified by indole, triple sugar iron, urease oxidase, and catalase tests. The resistance patterns of identified bacterial clinical isolates were confirmed using selective antibiotics (Oxoid, Basingstoke, Hampshire, UK) by employing the Kirby-Bauer disk diffusion technique as per the criteria set by CLSI [20, 21]. For Gram-positive bacteria antibiotics such as penicillin (10 *μ*g), chloramphenicol (30 *μ*g), tetracycline (30 *μ*g), vancomycin (30 *μ*g), erythromycin (15 *μ*g), gentamicin (10 *μ*g), and ciprofloxacin (5 *μ*g) were used. For Gram-negative bacteria ampicillin (10 *μ*g), piperacillin (100 *μ*g), ceftriaxone (30 *μ*g), cefepime (30 *μ*g), gentamicin (10 *μ*g), tetracycline (30 *μ*g), azithromycin (15 *μ*g), chloramphenicol (30 *μ*g), ciprofloxacin (5 *μ*g), and meropenem (10 *μ*g) were used. For the antimicrobial assay, inoculums were prepared by picking pure colony from nutrient agar with a sterile wire loop and suspended in sterile normal saline. The density of suspension to be inoculated was determined by comparison with opacity standard on McFarland 0.5 barium sulphate solution. The test organisms were uniformly swabbed over the Mueller-Hinton agar surface and exposed to a concentration gradient of antibiotic diffusion from antibiotic-impregnated paper disk into the agar medium and then at 35–37°C for 24 hrs. For fastidious bacteria, *S. pyogenes* and *Campylobacter* spp. antibiotic susceptibility testing was done in Mueller-Hinton agar supplemented with 5% sheep blood. Diameters of the zones of inhibition around the disk were measured to the nearest millimeter using a ruler and categorized as sensitive, intermediate, and resistant according to the standardized table described in Clinical and Laboratory Standards Institute. Bacteria showing resistance to two classes and above of antibiotics were inferred as MDR. List of MDR clinical isolates and corresponding antibiotic resistance patterns are shown in Table 1.

### 2.3. Agar Diffusion Assay

Aliquots (5 mg/mL) of the extracts were prepared with the solvent and their antimicrobial activity was further tested against clinical isolates according to the agar diffusion method described by Manilal et al. [15]. The assay was carried out in Mueller-Hinton agar (Himedia, India). Agar plates were prepared as per the instructions of the manufacturer. Overnight broth culture of the respective test organisms was swabbed on three axes onto the agar with a sterile cotton swab. Then, wells (5 mm in diameter) were made on agar plates by using a sterile cork borer. The resultant wells in triplicate were filled with 100 *μ*L of the plant extract. A well with EtOH was taken as a negative control. Petri plates were then incubated for 24 hrs at 37°C with the exception of *S. pyogenes* and *Campylobacter* sp. (inoculated in Mueller-Hinton blood agar and incubated at 37°C in 5% CO_2_ for 24 hrs). The inhibitory activity was measured by calculating the area of the inhibition zone on three axes. The extent of antimicrobial activities in this study is expressed in terms of area of inhibition zone: <50.24 mm^2^, inactive; 50.24–130.63 mm^2^, slightly active;132.66–254.34 mm^2^, moderately active; >254.34 mm^2^, highly active.

### 2.4. Determination of Minimum Inhibitory and Minimum Bactericidal Concentrations

The broth dilution method was employed to determine the minimum inhibitory concentration (MIC) of the crude plant extract. It was determined against the highly sensitive test strains of meat-borne bacterial pathogens such as *S. aureus*, *E. coli*, and *Salmonella* sp. The dried EtOH extract, aliquoted in phosphate-buffered saline (pH 7.2), was used as the test solution. The dosing range of plant extracts was computed by a factor of 2 (antilog 0.3) to obtain a final value ranging between 2000 and 32000 *µ*g per mL in nutrient broth. Afterwards, each tube was inoculated with 100 µL of a fresh overnight culture of the appropriate bacterial isolate and incubated at 37°C for 24 hrs. Based on the preliminary experiments, the concentration range of the plant extract was further narrowed to obtain the specific MIC value (2000 and 32000 *µ*g L). MICs were recorded as the lowest concentration that prevented the visible growth as indicated by the absence of turbidity in line with the control. To measure the minimum bactericidal concentration (MBC), MIC cultures were seeded (10 *µ*L) on Mueller-Hinton agar and incubated for 24 hrs at 37°C. MBC was defined as the concentration which exhibited no growth of the colonies compared to the culture of the initial inoculum, of the same strain. Plant extract is considered as bactericidal if the ratio of MBC/MIC is ≤ 2, and otherwise as bacteriostatic. A ratio, ≥16, hints at the ineffectiveness of the extract [15].

### 2.5. Gas Chromatographic and Mass Spectroscopic Analysis

Shimadzu QP-2010 GC-MS system equipped with a capillary column (inner diameter 0.25 mm and length 30 m) was used to analyse the constituents of the EtOH extract of *R. officinalis* [15]. The temperature of the GC oven was kept at 100 °C for two minutes and was further programmed to 280 °C at the rate of 10 °C/min and then kept at 280°C for 13 min. The split ratio was 1: 25 and the injection volume was 2 *μ*L. The injection port and detector port temperatures were 200°C and 240°C, respectively. The GC-MS electron ionization mode was 150 eV. The mass scanned was between m/*z* 20–500 amu (70 ms accumulation time). The peaks of the gas chromatogram were subjected to mass spectral analysis. The active constituents were identified based on the retention indices and by the comparison of mass spectra with the National Institute of Standards and Technology library of mass spectral data.

### 2.6. FT-IR Analysis

Functional groups of major components present in the crude EtOH extract were analysed and identified by recording the spectra between 400 and 4000 cm^−1^ (FT-IR spectrometer; Thermo Fisher).

### 2.7. UV-Visible Spectroscopy

*λ *_max_ of the crude extract in EtOH was measured using UV-visible spectroscopy (Shimadzu, software; Probe 2.50. Lamp; single beam).

### 2.8. Data Analysis

The data are described as mean ± standard deviation (S.D.) using SPSS for Windows version 20 (Statistical Package for Social Services, Chicago, IL, USA).

## 3. Results

### 3.1. Overall Antimicrobial Activity

**Figure 1:**
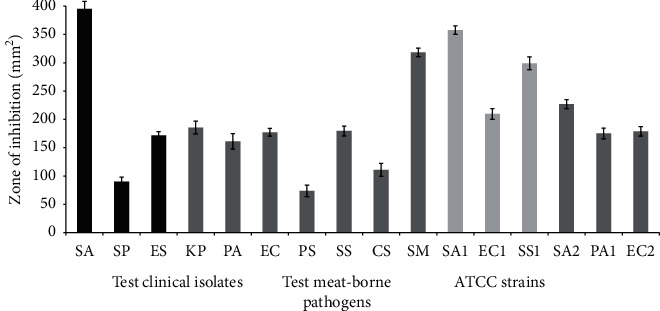
Antibacterial activities of EtOH extract of *R. officinalis* against clinical and meat-borne bacterial isolates. The activities are presented by the inhibition zone area of each tested strain. Clinical isolates: SA: *S. aureus*; SP: *S. pyogenes*; ES: *Enterococcus* sp.; KP: *K. pneumoniae*; PA: *P. aeruginosa*; EC: *E. coli*; PS: *Proteus* sp.; SS: *Shigella* sp.; CS: *Campylobacter* sp.; SM: *Salmonella* sp.; meat-borne pathogens: SA1: *S. aureus*; EC1: *E. coli*; SS1: *Salmonella* sp.; ATCC strains: SA2: *S. aureus*; PA1: *P. aeruginosa*; EC2: *E. coli*.

Fresh leaves of *R. officinalis* were extracted using EtOH in a mortar and pestle. The resultant extract was screened for the antimicrobial assay against a battery of type culture isolates and MDR clinical isolates as well as strains of meat-borne pathogens (Figure 1). EtOH extract successfully prevented the growth of both Gram-positive and Gram-negative clinical isolates. The extract displayed an area of inhibition ranging between 73.8 ± 10.05 and 395 ± 13.2 mm^2^. In the case of Gram-positive bacteria, the highest and moderate inhibitory values of 395 ± 13.2 ^2^ and 171.8 ± 6.73, respectively, were displayed against *S. aureus* and *Enterococcus* sp. However, the inhibitory value against *S. pyogenes* was found to be much lower (90 ± 8 mm^2^). In the case of the Gram-negative isolates, the highest inhibition area of 318.3 ± 7.6 mm^2^ was displayed against *Salmonella* sp. Moderate levels of activities were shown against *Shigella* sp. (179.6 ± 8.5 mm^2^), *Klebsiella pneumoniae* (185.52 ± 11.2 mm^2^), *P. aeruginosa* (161.13 ± 13.8 mm^2^), and *E. coli* (177.2 ± 6.9 mm^2^). On the other hand, lower inhibitory values were only demonstrated against *Proteus* sp. (73.8 ± 10.05 mm^2^) and *Campylobacter* sp. (111 ± 11.5 mm^2^). It has been found that the activity of the extract was moderate against type culture isolates, *P. aeruginosa* (175.14 ± 9.4 mm^2^), *S. aureus* (226.9 ± 8.1 mm^2^), and *E. coli* (178.26 ± 8.2 mm^2^) (supplementary file). Likewise, the EtOH extract of *R. officinalis* exhibited a broad spectrum of antibacterial activity against meat-borne pathogens, corresponding to an area of inhibition ranging from 209.6 ± 9.5 to 357.6 ± 7.5 mm^2^. However the extract was least active against *E. coli* (209.6 ± 9.5 mm^2^).

### 3.2. MIC and MBC

MIC, MBC, and MBC/MIC values are presented in Table 2. The determined MIC values of *R. officinalis* correspond to the range of 4. 10^3^ to 32. 10^3^ *µ*g/m/L and the concomitant MBC values are registered in the range of 8. 10^3^ to 32. 10^3^ *µ*g/mL. The lowest MIC and MBC values (4. 10^3^ and 8. 10^3^ *µ*g/mL) were recorded against Gram-positive isolates, *S. aureus*. However, the highest MIC and MBC values (16. 10^3^ and 32. 10^3^ *µ*g/mL) were displayed against Gram-negative isolates, particularly the *E. coli.* The overall results revealed that antibacterial constituents exist in the extract of *R. officinalis*.

### 3.3. Chemical Constituents of the EtOH Extract of *R. officinalis*

To identify the chemical constituents responsible for antimicrobial activity, crude EtOH extract of *R. officinalis* was subjected to GC-MS, FT-IR, and UV-vis spectral analysis. In the GC-MS analysis, a total of fourteen prominent peaks were observed with retention times as presented in Table 3. The major constituents as per the peak area percentage analysed are camphor (13%), endo-borneol (13%), alpha-terpineol (12%), hydroxyhydrocaffeic acid (13%), and genkwanin (26%). Of that, genkwanin (O-methylated flavone), alpha-terpineol, and camphor were known to be prominent antimicrobial compounds. The major compound detected was genkwanin, a substituted flavone (5, 4-dihydroxy-7-methoxy flavone). In the FT-IR spectrum, presence of aromatic hydroxyl groups (-OH) in the compound is evidenced by a broad band around 3345 cm^−1^. Another important characteristic sharp band observed is 1660 cm^−1^ (C=O group). Medium intense bands are observed around 1466 cm^−1^, 1372 cm^−1^, 827 cm^−1^, and 784 cm^−1^ which are due to the presence of C-C stretching, C-H bending, and C-H out-of-plane bending, respectively. Ether stretching bands are observed at 1340 cm^−1^, 1200 cm^−1^, and 1180 cm^−1^. UV_max_ in EtOH corresponds to 267, 300, and 333 nm due to the flavonoid moiety in the molecule. The presence of genkwanin is confirmed by the existence of a deprotonated molecular ion, [M-H]^−^ of m/*z* 283, in the negative ion mode (100 to 1000 amu and with 200 ms accumulation time) and this corresponds to an elemental composition of C_16_H_12_O_5_. A series of characteristic fragment ions at m/*z* 268, 240, 165, 151, and 117 also confirm the presence of genkwanin molecule, the predominant fragment being the one with m/*z* 268 which has a stable structure (resulting from the loss of -CH_3_ from the deprotonated molecular ion). A Retro-Diels Alder (RDA) cleavage of genkwanin molecule resulted in the formation of C_7_H_3_O_4_ (m/*z* 151) and C_8_H_5_O (m/*z* 117) (Table 4).

Next series of major compounds include camphor, endo-borneol, alpha-terpineol, and hydroxyhydrocaffeic acid with more or less equal percentage composition (between 12 and 13%). The presence of camphor is evidenced in the FT-IR spectrum which showed a broad absorption around 2995 cm^−1^ (due to the sp^3^ C-H stretching), 1744 cm^−1^ (C=O stretching), and 1477 cm^−1^ due to isopropyl group. UV_max_ in EtOH corresponds to 290 nm which also confirms the presence of camphor. Additional evidence for the presence of camphor molecule is obtained from the fragmentation pattern in the mass spectrum; the m/*z* 152 (C_10_H_16_O^+^), 108 (C_8_H_12_^+^), 95 (C_7_H_11_^+^), 81 (C_6_H_9_^+^), 69 (C_4_H_5_O^+^), 55 (C_4_H_7_^+^), 41 (C_3_H_5_^+^), 39 (C_3_H_3_^+^), and 27 (C_2_H_3_^+^) values correspond to the fragmentation pattern of camphor molecule (Table 4).

The presence of endo-borneol in the crude extract is confirmed by FT-IR spectrum which exhibited a broad absorption peak at 3352 cm^−1^ due to hydroxyl group and a strong band at 1055 cm^−1^ due to the C-OH stretching vibration. From the mass spectral analysis of the crude extract, the presence of borneol is confirmed by the existence of fragments/molecules with m/z 136 (elimination of water from endo-borneol), m/z 95, and m/z 110, the former due to the elimination of –C_3_H_5_ entity from the dehydrated borneol and the latter by the elimination of –C_2_H_2_. At the same time, fragments with m/z 108 were also observed which might have occurred due to the elimination of C_2_H_4_ from the dehydrated endo-borneol (m/z 136 via RDA). In addition there exists a fragment with m/*z* 93, which corresponds to C_7_H_9_ + (via the elimination of CH_3_) (Table 4).

The presence of alpha-terpineol is confirmed by FT-IR spectrum which showed stretching of -OH group at 3394 cm^−1^, cluster of carbon with double bonds (C=C) of alkenes, at 1674 cm^−1^, band at 1373 cm^−1^, due to the presence of -CH_3_ group, and –C-O- bond of tertiary alcohol at 1134 cm^−1^. UV-visible *λ*_max_ in EtOH of this compound corresponds to 225 nm as is observed in the spectrum of the crude extract. The MS analysis of the corresponding peak from GC produced m/z 154 (molecular ion peak of alpha-terpineol), followed by the breaking away of –OH forming m/z 136 and then the elimination of –CH_3_ resulting in fragment with m/z 121. Further elimination of C_2_H_4_ from the aforementioned fragment produces the entity with m/z 93 and again the release of C_7_H_12_ produced a final and stable fragment with m/z 59. This characteristic fragmentation confirms the presence of alpha-terpineol (Table 4).

Another major compound found in the crude extract is hydroxyhydrocaffeic acid. The FT-IR analysis of the extract revealed the presence of stretching vibration of carboxyl group, -COOH, at 2847 cm^−1^ (corresponding to OH), carbonyl vibration at 1668 cm^−1^, and deformation vibration of –OH group at 1460 cm^−1^. Also the –COOH group exhibited medium intensity bands at 1169 cm^−1^ and 698 cm^−1^ due to –C-OH- group. The –OH group attached to aromatic ring produced vibrations at 3356 cm^−1^ and there exists C-OH vibration at 1219 cm^−1^. UV-visible *λ*_max_ in EtOH of this compound corresponds to 280 nm. The MS analysis showed [M-H]^−^ ion at m/z 197 in the negative ion mode proving the presence of C_9_H_15_O_5_, hydroxyhydrocaffeic acid. The fragmentation series obtained in the mass spectral analysis include ions having m/z 179 and 135 which resulted from the elimination of H_2_O and the subsequent expulsion of CO_2_, respectively, from the [M-H]^−^ ion. The fragment ion at m/z 73 was formed after the loss of –CHOH and –COOH from the benzyl carbon of the parent molecule (Table 4).

## 4. Discussions

In developing countries, the prevalence of bacterial infections is rising due to various factors such as the HIV/AIDS pandemic, poor hygiene, overcrowding, malnutrition, and scarcity of potable water. Above all, the resistance to conventional antimicrobials worsens the problem. The use of natural antimicrobial agents could represent a low-cost alternative therapy especially for rural communities. In Ethiopia, traditional medicine is an integral part of the indigenous culture and serves as a major public health system [22].

It has been reported that there are ample chances of finding biological activity of plants with recorded medicinal uses, instead of opting for the random selection method [23]. Besides, the selection of crude plant extracts for screening is more rapid and practical than utilizing isolated pure compounds [23]. Phytochemical analyses of plant extracts having antimicrobial activity could provide a platform for discovering new molecules with potentiality to serve as suitable leads for the development of new antibiotics, eventually helping in combating MDR bacteria, either through further derivatization or by total synthesis routes.

This study is part of an ongoing screening program aiming at the discovery of Ethiopian medicinal plants with antimicrobial activities [24–26]. Despite the medicinal and extensive culinary uses of *R. officinalis* in different regions of Ethiopia, no previous studies have evaluated its antimicrobial activity against MDR clinical isolates as well as meat-borne bacterial pathogens. In a previous study, we have revealed the pronounced antimicrobial activity of extracts of *R. officinalis* against the biofilm-forming MRSA [15]. The present study is mainly focused on comprehensively evaluating the antimicrobial activity of EtOH extract of this plant against the aforementioned battery of bacteria. It is envisaged that clinical isolates can often reflect the contemporary genetic profile of microorganisms in local healthcare settings. And these genetic differences are imperative while evaluating the relevance of medicinal plants. In general, the EtOH extract of *R. officinalis* efficiently repressed the growth of all the ten tested MDR clinical isolates and the three meat-borne pathogens to varied degree. The broad antimicrobial activity exhibited by the extract could be due to the presence of soluble bioactive constituents of myriad chemical nature. It is previously reported that the solubility of the antimicrobial constituents could be influenced by the nature of solvent used for the extraction and its polarity [27].

The activity was noteworthy against *Salmonella* sp. and *S. aureus* suggesting the wide spectrum nature towards both Gram-positive and Gram-negative clinical isolates. Broad-spectrum antimicrobial activity of several compounds from *R. officinalis* from other parts of the world is well-documented in the literature [28–31]. However, the majority of these studies are merely done in standardized strains or clinical isolates using essential oil and not using the extract. It can be speculated that the number of constituents and the chemical diversity could be superior in the case of the latter. Our study is the first to test an EtOH extract prepared from the tender foliage of the plant against MDR clinical isolates, ATCC bacterial isolates, and meat-borne pathogens. The latter group of pathogens are extremely important in an Ethiopian context, because plenty of dishes comprising raw meat are consumed throughout the nation along with the tender leaves of *R. officinalis.*

However, it was found that the activity of the extract was considerably lower towards Gram-negative isolates such as *Proteus* sp. and *Campylobacter* sp. This could be due to the structural variances in the cell membranes of Gram-negative bacteria compared to their Gram-positive counterparts. It is well known that the double membrane in Gram-negative bacteria is associated with ineffective membrane penetration by bioactive molecules and greater difficulty for them to reach active sites [32]. Besides, the antibacterial potency can be influenced by various other factors such as evaporation, solubility, and diffusion rate of the bioactive components. Only a lower level of activity was observed in the case of the Gram-positive isolate, *S. pyogenes*, and this has to be studied in detail. In our study, the extract of *R. officinalis* showed a moderate level of activities against the ATCC bacterial isolates.

The actual mechanism of antibiosis is not explored in this study. However, it could be envisaged that the antimicrobial efficacy of rosemary extract could be attributed to the presence of broad-spectrum bioactive metabolites that might interact with the cell membrane, causing changes in genetic material and nutrients which alter the electron transport mechanism. Also the leakage of cellular components, changes in the rate of fatty acid formation, and interaction with protein membranes can cause the loss of functionality and structural integrity of the bacterial cell. This would substantiate the efficacy of bioactives [31, 33]. Further in-depth studies are required to have a conclusion. Culinary herbs and spices in general are known to contain myriads of phytochemicals with medicinal properties [34]. Regarding the meat-borne pathogens, the activity of the plant extract was found to be higher against *S. aureus* followed by *Salmonella* sp. A thorough literature survey indicates that antimicrobial activity of other plant species against meat-borne pathogens are reported by several researchers [35, 36]. Besides, the ratio of MBC/MIC against three meat-borne pathogens was found to be ≤ 4 and therefore the mechanism of antibiosis of plant extract can be inferred as bactericidal [15, 26].

Secondary metabolites are long been providing important drug leads for various infectious diseases. Based on GC-MS, FT-IR, and UV-vis spectroscopic studies of EtOH extract, major secondary metabolites from *R. officinalis* were identified. It was observed that the major compounds are camphor, endo-borneol, alpha-terpineol, hydroxyhydrocaffeic acid, and genkwanin (26%).

The major compound according to the peak area was found to be genkwanin, which is a methyl substituted flavone. This compound is reported as one of the components in the extract of *R. officinalis* by various researchers but to different extents. Antitumor, antioxidant, and immune-modulatory activity of genkwanin and its hydroxy derivatives are studied recently *in vitro* and *in vivo* [37]. Genkwanin is the representative marker for quality control of Genkwa flos (the dried flowers of *Daphne genkwa*) in China Pharmacopoeia. There are several reports showing the antimicrobial and lower cytotoxic activities of genkwanin and its related flavonoids [12, 37, 38].

Another important constituent found in our study is alpha-terpineol which is also an antibacterial monoterpene and known to exist in *R. officinalis* [39]. Endo-borneol is a secondary alcohol of the bicyclic terpene group and is found in various plants including *R. officinalis* [40]. It is known as an agent which improves blood circulation and is used for the treatment of bronchitis, coughs, and colds and for reducing pains and swellings. It is also used as an insect repellent. Hydroxyhydrocaffeic acid or ‘danshensu' belongs to the class of organic compounds known as phenyl propanoic acid and is an extremely weakly basic compound [41, 42]. This compound has been detected in *R. officinalis* earlier and is a potential biomarker for the consumption of food items. Camphor was also found in appreciable quantity in *R. officinalis* as per our analysis and is a monoterpene [8]. Other than these listed major compounds, there are more than ten compounds such as eucalyptol, bornyl acetate, cis-myrtanol, substituted phenols, ferruginol, caryophyllene, caryophyllene oxide, and octadecanoic acid [8]. There is a probability of synergistic interaction by all these compounds with regard to the antimicrobial activity of *R. officinalis.* However we could not find some of the typical compounds usually found in *R. officinalis* such as carnosic acid, carnosol, rosmadial, rosmaridiphenol, and rosmarinic acid which are compounds with known bioactivities [12, 43]. Moreover, triterpenes such as ursolic acid and augustic acid were also found absent [12]. It could also be inferred that these compounds are bactericidal and/or bacteriostatic influencing the lag time, rate of bacterial growth, and also the maximum growth [44].

Despite the existence of genetic control, gene expression, and genotypes, the total content and relative proportions of secondary metabolites in plants may fluctuate over time and space and even in the case of the same species found in different locations [45]. The quantity, composition, and ratio of secondary metabolites are influenced by a number of internal and external factors, such as the age of the plant, pollution, evolution, climate, type of soil, type of plant materials (leaves, flowers, etc.), altitude, precipitation, or stress conditions that may inhibit or trigger the synthesis of specific compounds with bioactivities [12, 45, 46]. In addition, even for the same soil type, slight shift in its pH and composition can also have an influence [47]. Other factors affecting antimicrobial activities are the time of harvest, storage temperatures, the plant organ being extracted, the type of solvent used, extraction methods and the period, and even extraction conditions [43].

## 5. Conclusions

To the best of our knowledge, this is the first report dealing with the antibacterial activity of the extract of *R. officinalis,* particularly against MDR clinical isolates and meat-borne pathogens. The mechanism of antibiosis of plant extract is inferred to be bactericidal. GC-MS, FT-IR, and UV-vis spectral analyses revealed the presence of major compounds such as genkwanin (26%), camphor (13%), endo-borneol (13%), and hydroxyhydrocaffeic acid (13%). Overall results of the present study conclude that *R. officinalis* could be an excellent source of antimicrobial agents for the management of drug-resistant bacteria as well as meat-borne pathogens. The possibility of using this plant extract as an antimicrobial agent has far-reaching consequences as it provides inspiring leads for future research. A detailed study is being underway on various aspects of the mechanism of antibiosis and toxicity of compounds isolated from this plant.

## Figures and Tables

**Table 1:** Panel of MDR clinical and meat-borne bacterial pathogens used for antibacterial assay.

Specimens	Types of MDR clinical isolates	Antibiotic resistance patterns
Wound	*S. aureus*	Penicillin, gentamicin, erythromycin, tetracycline, and ciprofloxacin
	*K. pneumoniae*	Piperacillin, ceftriaxone, cefepime, azithromycin, and meropenem
	*P. aeruginosa*	Piperacillin, cefepime, meropenem, gentamicin, and ciprofloxacin

Throat	*S. pyogenes*	Tetracycline, ceftriaxone

Urine	*Enterococcus* sp.	Penicillin, erythromycin, and ciprofloxacin
	*E. coli*	Penicillin, tetracycline, ampicillin, ceftriaxone, and co-trimoxazole
	*Proteus* sp.	Ampicillin, gentamicin, ciprofloxacin, co-trimoxazole, and ceftriaxone

Stool (diarrhoea)	*Shigella* sp.	Ampicillin, ciprofloxacin, co-trimoxazole, gentamicin, ceftriaxone, and erythromycin
	*Campylobacter* sp.	Ampicillin, chloramphenicol, ciprofloxacin, co-trimoxazole, gen; gentamicin, erythromycin, tetracycline, and ceftriaxone
	*Salmonella* sp.	Ampicillin, chloramphenicol, ciprofloxacin, co-trimoxazole, gentamicin, erythromycin, and ceftriaxone

Meat-borne pathogens	*S. aureus*	—
	*E. coli*	—
	*Salmonella* sp.	—

ATCC reference strains	*S. aureus* (25923)	—
	*E. coli* (27853)	—
	*P. aeruginosa* (27853)	—

**Table 2:** Minimum inhibitory concentration, minimum bactericidal concentration, and MBC/MIC of EtOH extract of *R. officinalis*.

Test organisms	*R. officinalis*		
	MIC (*µ*g/mL)	MBC (*µ*g/mL)	MBC/MIC
*S. aureus*	4. 10^3^	8. 10^3^	2
*E. coli*	8. 10^3^	16. 10^3^	2
*Salmonella* sp.	8. 10^3^	32. 10^3^	4

**Table 3:** The phytoconstituents identified from the EtOH extract of *R. officinalis* by GC-MS analysis.

RT	Name of the compound	MF	MW	PA (%)	Functional group
5.09	Eucalyptol	C_10_H_18_O	154	1.5	Cyclic ether (monoterpenoid)
8.17	Camphor	C_10_H_16_O	152	11.8	Ketones (terpenoid)
9.04	Borneol	C_10_H_18_O	154	12	Bicyclic terpene (trimethyl with OH group)
9.79	Alpha-Terpineol	C_10_H_18_O	154	12	Monoterpene alcohol
11.05	cis-Myrtanol	C_10_H_18_O	154	2	Dimethyl bicyclo heptan-2-methanol
11.92	Bornyl acetate	C_12_H_20_O_2_	196	2	Ester
14.06	Camphene	C_10_H_16_	136	2	Bicyclic terpene
15.17	Caryophyllene	C_15_H_24_	204	3	Bicyclic sesquiterpene
17.99	Hydroxyhydrocaffeic acid	C_9_H_10_O_5_	198	12.5	Hydroxy acid
19.17	Phenol, 2, 4-bis (1,1-dimethyl)	C_14_H_22_O	206	2	Dimethyl substituted phenol
20.8	Caryophyllene oxide	C_15_H_24_O	220	3	Oxidic bicyclic sesquiterpene
24.8	Genkwanin	C_16_H_12_O_5_	284	26	O-Methylated flavone
31.3	Propanoic acid, 3-mercapto-dodecyl ester	C_15_H_30_O_2_S	274	6.5	Sulphur containing ester
34.7	Octadecanoic acid	C_18_H_36_O_2_	284	4	Fatty acid
36.6	Ferruginol	C_20_H_30_O	286	2	Phenolic monoterpene

**Table 4:** Spectral identification of five proposed major compounds.

Name of the major compound	Fragments (m/*z* values, amu)	IR vibrations (cm^−1^)	*λ *_max_ (nm)
Genkwanin	[M-H]^−^, 283, 268, 240, 165, 151, and 117	3345,	267, 300 and 333
		1660,	
		1466, 1372, 827, 784	
		1340, 1200, 1180	

Camphor	152, 108, 95, 81, 69, 55, 41, 39, and 27	2995,	290
		1744,	
		1477	

Endo-borneol	136, 110, 108, 95, and 93	3352,	—
		1055	

Alpha-Terpineol	154, 136, 121, 93, and 59	3394,	225
		1674,	
		1373,	
		1134	

Hydroxyhydrocaffeic acid	[M-H]^−^, 197, 179, 135, 73	3356,	280
		2847,	
		1668,	
		1460,	
		1169, 698	

## Data Availability

The data used to support the findings of this study are included within the article.

## Supplementary Materials

Supplementary Materials Area of inhibition zone obtained by EtOH extract of *R. officinalis* against type culture strains of *S. aureus* (ATCC 25923) and *E. coli* (ATCC 25922).
