# Supplementary material for: Antibacterial Activity of Rosmarinus officinalis against Multidrug-Resistant Clinical Isolates and Meat-Borne Pathogens
Source: Evid Based Complement Alternat Med. 2021 Apr 29;2021:6677420. doi: 10.1155/2021/6677420 (PMC8102098; doi:10.1155/2021/6677420)
Supplement: Supplementary Materials — Area of inhibition zone obtained by EtOH extract of R. officinalis against type culture strains of S. aureus (ATCC 25923) and E. coli (ATCC 25922). [file 6677420.f1.docx]

| 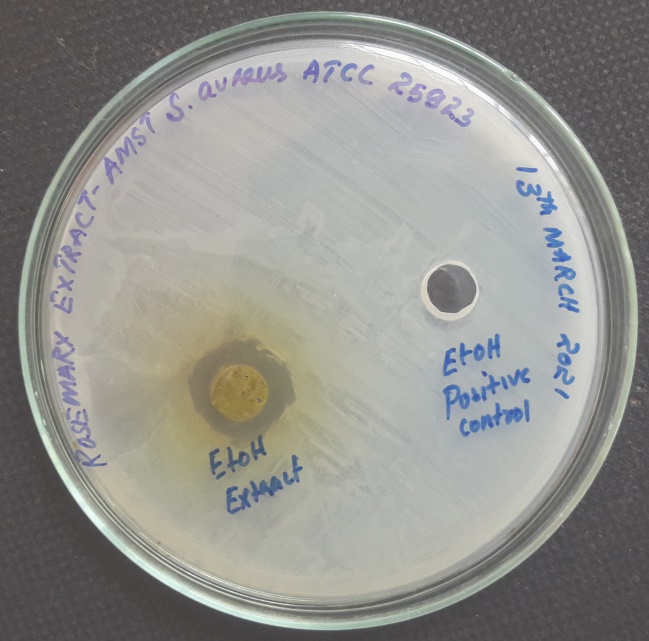 | 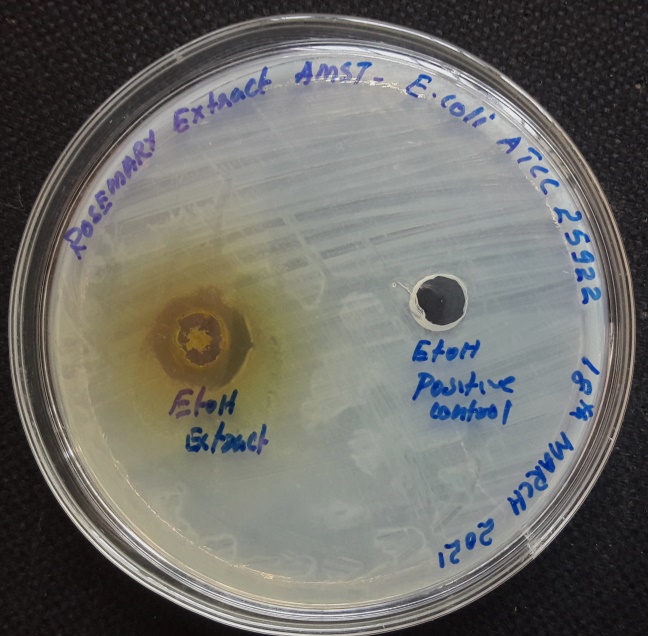 |
| --- | --- |
| Supplementary File. Area of Inhibition zone obtained by EtOH extract of *R. officinalis*  against type culture strains of *S. aureus* (ATCC 25923) and *E. coli* (ATCC 25922) | |
